# Conference report for the 2nd annual American Society for Intercellular Communication (ASIC) meeting, 2022

**DOI:** 10.20517/evcna.2022.43

**Published:** 2023-07-04

**Authors:** Ashley E. Russell, Michael W. Graner, Shilpa Buch

**Affiliations:** ^1^Department of Biology, School of Science, Penn State Erie, The Behrend College, Erie, PA 16563, USA.; ^2^Department of Neurosurgery, University of Colorado Anschutz Medical Campus, Aurora, CO 80045, USA.; ^3^Department of Pharmacology and Experimental Neuroscience, University of Nebraska Medical Center, Omaha, NE 68198, USA.

The second annual meeting of the **American Society of Intercellular Communication (ASIC)** was held in Potomac, Maryland on October 12th-15th, 2022. The hybrid format of this meeting was similar to the previous year, with both in-person and virtual attendees. The vast majority of conference participants attended in person, while remote presenters submitted pre-recorded oral and poster presentations that were shared via Zoom, and live Q&A sessions were held, facilitating dynamic discussions among participants from both modalities.

This year’s meeting drew more attendees than the previous year, with 152 participants, and was supported by 11 sponsors, including Alpha Nano Tech, BioTrac, Ceres Nanosciences, Extracellular Vesicles and Circulating Nucleic Acids (EVCNA), Izon, Kimera Labs, Kinetic River, ONI Bio, Particle Metrix, Unchained labs, and Virogny Biosciences. This three-day meeting had 61 talks (47 of which were given in person), a Grant writing workshop, and 33 posters presented during a session on the evening of day two.

**Day 1 of Meeting:** The meeting started with an informative pre-program NIH workshop moderated by Shilpa Buch (University of Nebraska Medical Center) and Amanda Brown (Johns Hopkins University). Several NIH program officers, including Christine Happel (Program Director, National Center for Advancing Translational Sciences/NIH) Anna Sadusky (Program Director, Division of Kidney, Urologic and Hematologic Disease, National Institute of Diabetes, and Digestive and Kidney Diseases/NIH), Aniruddha Ganguly (Program Director, Office of the Associate Director, Cancer Diagnosis Program, Division of Cancer Treatment & Diagnosis, National Cancer Institute/NIH), and Martha Lundberg (Program Director, Advanced Technologies and Surgery Branch, National Heart, Lung and Blood Institute/NIH) gave presentations focusing on various funding mechanisms related to extracellular vesicles (EVs) and extracellular RNA (exRNA), research priorities in various subject areas, and several grant writing tips.

This funding-focused session was followed by a series of scientific talks, the first of which was the keynote lecture given by Shilpa Buch (University of Nebraska Medical Center), whose talk focused on the role of morphine-mediated astrocytic senescence. Astrocytic senescence induced by morphine *in vitro* was found to be mediated by a long non-coding (lnc)RNA. This work also suggested that astrocyte-derived EVs (ADEVs) may be carriers of senescence cargo which could be taken up by neurons, leading to synaptodendritic injury and neuronal senescence. *In vivo* evidence showed that morphine-administered mice exhibited an aging phenotype and cognitive deficits. Collectively, these data suggest that lncRNAs may play a role in mediating astrocytic senescence and ADEVs may, in part, mediate synaptodendritic injury associated with aging.

The next talk was by Nicole Noren Hooten (National Institute on Aging/NIH), which focused on the role of EVs in aging and age-related conditions.

Next, Hameeda Sultana (University of Tennessee) presented work on the exosome-mediated transmission of flaviviruses to vertebrate hosts from tick and mosquito saliva. When these insects bite a host, inflammatory and wound healing pathways are activated. Interestingly, these pathways can be modulated by tick-derived salivary EVs delivered to the host at the bite site, and these EVs have the capacity to bind to skin barrier cells, including keratinocytes. Further, tick cells treated under various salt, pH, and temperature conditions release EVs that can modulate viral transmission and replication in human skin cells.

Chioma Okeoma (Stony Brook University Renaissance School of Medicine) presented next, focusing on human immunodeficiency virus (HIV) and drugs of abuse, specifically Δ9-THC. Using the simian immunodeficiency virus (SIV) non-human primate model of HIV, it was found that during infection, the basal ganglia release pathogenic EVs associated with neuroinflammation with distinct microRNA (miRNA) profiles. Treatment with Δ9-THC was shown to modulate basal ganglia-derived EV miRNAs, many of which have predicted targets associated with inflammation, immune regulation, and cell death pathways. SIV basal ganglia EVs were also shown to alter astrocytes and microglia, but Δ9-THC exposure reduces these changes.

The next presenter was Ursula Sandau (Oregon Health & Science University), who shared recent data focusing on the relationship between the clinical features of methamphetamine use disorder and EV miRNA profiles. Plasma EVs from humans with active methamphetamine use were found to contain miRNAs that correlated to a number of variables, including frequency of methamphetamine use, age of onset, and lifetime exposure, as well as neuropsychiatric function, (anxiety, memory, and pain), inflammation, and CNS injury compared to healthy controls. These addiction-relevant EV miRNAs could be potential targets for the creation of novel therapeutics for treating methamphetamine use disorders.

Amanda Brown (Johns Hopkins University) gave the next presentation on studying HIV infection by use of human microglial cells. This work demonstrated that bone marrow myeloid progenitor cells could be isolated from adult human blood and differentiated, *in vitro*, into microglial-like cells. These cells expressed a number of common microglial markers after approximately one week in culture, and several of these markers persisted for three weeks at both the gene and protein levels. Importantly, these cells were cultured under serum-free conditions and expressed CD45, making them ideal candidates for studying both EV biogenesis and HIV infection.

The next talk was given by Carlos Osterman (Ponce Health Sciences University), which focused on lipid metabolism and prostate cancer. Prostate cancer is the 5th leading cause of cancer-related deaths worldwide and the 1st in Puerto Rico. Prostate cancer tumors undergo shifts in their metabolic profiles, including increased lipid metabolism and fatty acid synthesis, which allows them to escape existing therapies. In this work, cell-based models of prostate cancer therapy resistance were used for metabolic profiling and found that pathways utilized for building fatty acids and lipids were upregulated in these cells. One enzyme, fatty acid synthase, has been shown to be upregulated by cancer progression and metastatic stage. Further, this enzyme was also identified in EVs released by prostate cancer cells. Incubation of EVs derived from drug-resistant prostate cancer cells with macrophages resulted in a shift towards M2 polarization, indicating that these EVs may influence the tumor-immune microenvironment.

The next presentation was given by Gurudutt Pendyala (University of Nebraska Medical Center). In this work, nanoplasmonics-based single EV analysis was used to identify differences in brain-derived EVs (BDEVs) from mice with acute or chronic methamphetamine use. First, EVs were assessed via quantitative mass spectrometry-based proteomics and revealed distinct profiles for the two substance use paradigms. Then, EVs were analyzed through the nanoplasmonics-based single EV multiplexing platform, which validated the mass-spec proteomic data at the single-EV level. Together, these platforms indicate that specific pathways in the CNS are activated in each of the substance use paradigms.

Next, David Greening (Baker Heart and Diabetes Institute, Australia) presented recent work on the surface proteome of EVs. The membrane surface of an EV (the surfaceome) serves as the fundamental gateway between intra and extracellular signaling networks and dictates an EV’s capacity to communicate and interact with its environment. Understanding the surfaceome of EVs would provide insight into the biodistribution and diagnostic potential of EVs and allow for EV surface engineering to alter things such as uptake by modulating the expression of ligand receptors. Expanding our knowledge of the EV surfaceome will allow for a deeper understanding of EV biology, thereby expanding the utilization of EVs as diagnostic and therapeutic agents.

Eva Poveda (Galicia Sur Health Research Institute, Spain) gave the next presentation on the role of EVs in inflammation and cardiovascular disease (CVD) during HIV infection. Rates of CVD in people living with HIV (PLWH) tend to be higher than in the uninfected population, and CVD leads to a greater risk of stroke, myocardial infarction, and heart failure. A subpopulation of PLWH known as elite controllers spontaneously control HIV replication without antiretroviral therapy (ART); this can be transient or persistent but offers a model of functional cure. This work showed that plasma levels of EV-associated cytokines are different across a number of different conditions including persistent elite controllers, transient elite controllers, PLWH whose treatment plan includes ART, ART naïve PLWH, and healthy uninfected controls. These EV-associated cytokines may play a role in mediating the activation of inflammatory pathways and controlling HIV replication.

The last talk of the pre-program workshop was delivered by Gary Linz (Particle Metrix), who spoke about the importance of tools and technologies used to profile EVs, as there are many considerations that must be taken into account when deciding which type of particle counting platform to utilize for a specific experiment. Good results start with good samples and triplicate measurements should be made for each sample to ensure data accuracy.

The evening session was moderated by Julie Saugstad (Oregon Health & Science University) and Ursula Sandau (Oregon Health & Science University).

The keynote speaker for this session was Thomas Sudhof (Stanford University), who gave a presentation centered on trans-synaptic adhesion molecule signaling and its role in controlling postsynaptic membrane traffic. Extra and intracellular vesicle trafficking is critical for physiology, yet for decades it was not clear how these processes were mediated. Synapsins were thought to be the most important proteins in synaptic vesicles for neurotransmitter release, and a number of highly sophisticated experiments were required to elucidate its mechanism of action. For example, neurons with synapsin-1 deletion have approximately 1/3 fewer synaptic vesicles relative to wild-type neurons, and these neurons cannot sustain synaptic transmission to the same level as wild-type neurons during stimulus trains.

The next speaker of the evening session was Lance Liotta (George Mason University), whose talk focused on tumor-derived EVs found in interstitial fluid. As tumors increase in size, they exert higher interstitial fluid (IF) pressure, which is relieved via the lymphatics. The IF is rich with tumor-derived EVs, and this work demonstrated a novel approach for extracting *in vivo* IF EVs from a syngeneic murine cancer model without disrupting the tumor tissue histology. These EVs were found to be different than EVs isolated from *in vitro* cultured cancer cells, suggesting that the 3D environment affects EV profiles. Further, IF EVs were also found to contain full-length fission mitophagy proteins, indicating that these EVs can lend insight into the status of tumor cell mitochondrial biogenesis and destruction.

The next speaker in this session was Jonathan Geiger (University of North Dakota), who presented work on the endolysosomal stress response. The greater endolysosomal system plays a significant role in various cellular processes, including phagocytosis, antigen presentation and immune responses, cholesterol homeostasis, plasma membrane repair, mitochondrial division, mitophagy, exocytosis, and cell death. Its pathological relevance extends to aging and age-related neurological disorders, HIV-associated neurocognitive disorder (HAND), glioblastoma, and other neurogenerative disorders. Drugs that affect the endolysosomal system have been shown to affect the production, secretion, and content of EVs. Endolysosomes also act as master regulators of iron metabolism, and altering their pH has profound effects. The release of iron from endolysosomes increases iron levels in the cytosol and mitochondria, resulting in oxidative stress and cell death. Modulation of endolysosomal pH and iron release may be early events associated with EV release and may play a key role in the pathophysiology of various neurodegenerative diseases.

Jeff Franklin (Vanderbilt School of Medicine) gave the next presentation on the use of large-scale bioreactors for EV and non-vesicular extracellular particle production. Hollow-fiber bioreactors were employed to achieve a 20-fold increase in particle yield compared to traditional 2D cell culture growth. Interestingly, this work showed that EVs generated from hollow-fiber bioreactors and 2D cell cultures had similar proteomes. FPLC purification revealed distinct peaks for exomeres and supermeres, with the first peak containing membrane-associated proteins such as CD63, indicating an EV-enriched fraction. These findings underscore the potential of bioreactors in producing abundant secreted material for a number of applications.

The next talk was delivered by Michael Bukrinsky (George Washington University), which focused on EV-associated HIV Nef. The HIV accessory protein, Nef, was found to impact cholesterol metabolism and immune responses in macrophage and myeloid cells. Interestingly, less than 1% of Nef produced by Nef-transfected cells is associated with EVs. Isolation via ultracentrifugation revealed that the majority of Nef released by these cells is in the non-vesicular supernatant. Furthermore, EV-associated Nef was found to be preferentially located on the outside of EVs and is significantly more biologically potent than recombinant Nef.

Girish Neelakanta (University of Tennessee) next presented work on tick-derived EVs. The prevalence of several tickborne illnesses including Lyme disease, Rocky Mountain spotted fever, ehrlichiosis, and anaplasmosis, are on the rise. Anaplasmosis is caused by the bacterium *Anaplasma phagocytophilum*, which was found to induce EV production in tick cells. Interestingly, *A. phagocytophilum* type IV effector gene transcripts were found in EVs derived from tick and mammalian cells. These data collectively suggest that *A. phagocytophilum* infection may alter EV release and composition, and these EVs may be involved in the pathophysiology of infection.

The next presenter, Xandra Breakefield (Harvard University), shared recent work on EV communication in the brain. Glioblastoma-derived EVs were shown to be taken up by microglia and alter their ability to sense things around them, including the tumor itself and other pathogens. These EVs appear to serve a protective function that prevents the tumor from being detected by microglia. Glioma cells and their released EVs contain high levels of miR-21, and intracranial injection of glioma-derived GFP EVs into miR-21-null mice resulted in the transfer of these EVs to microglia and downregulation of miR-21 target genes. Work from the Breakefield lab further showed that neuron-derived EVs could spread in all directions within the brain and modify the physiologic function of neighboring cells, which has large implications for the role of EVs in the pathophysiology of numerous neurological diseases.

Ryan Flynn (Harvard University) gave the last talk of the evening, which focused on glycoRNAs. The major building blocks of cells can be glycosylated, creating glycoproteins, glycolipids, and glycoRNAs. GlycoRNAs are predominately formed from the attachment of sialylated, fucosylated *N*-glycans to RNAs. A number of RNA species have been found to be glycosylated, including small non-coding RNAs. The localization of glycoRNAs is primarily on the cell surface, which presents new opportunities for exploring the involvement of glycoRNAs in exRNA biology.

**Day 2 of Meeting:** The morning of the second day of the conference began with a session on heterogeneity and EVs, moderated by Lucia Languino (Thomas Jefferson University) and Heather Branscome (American Type Culture Collection; ATCC).

Lucia Languino (Thomas Jefferson University) kicked off this session with a keynote address focusing on the role of pro-tumorigenic EVs in cancer progression. Small EV (sEV) proteomics revealed differentially expressed proteins that are involved in cellular assembly and movement, inflammatory responses, and lipid metabolism. Integrins present in prostate cancer sEVs were shown to be functionally active upon uptake by macrophages and endothelial cells, and push macrophages towards the M2 phenotype, which may promote cellular reprogramming towards a pro-tumorigenic tumor microenvironment.

The next presentation was given by Qin Zhang (Vanderbilt School of Medicine) and described the functional role of supermeres. Supermeres are described as extracellular nanoparticles distinct from EVs and exomeres, which can be obtained via differential ultracentrifugation. Exomeres are separated from the supernatant after pelleting EVs, and similarly, supermeres are separated from the supernatant after pelleting exomeres. Supermeres were shown to have unique biodistributions and proteomic and RNA profiles, with clinical diagnostic potential.

Uta Erdbrügger (University of Virginia Health System) gave the next presentation on the role of EVs in hypertension (HTN). Approximately 1 in 3 Americans have HTN, which is a major risk factor for CVD and kidney disease. These patients have higher levels of circulating EVs, typically of endothelial or leukocyte origin, which correlate with disease severity and may reduce endothelial-dependent vasodilation in resistance arteries. Elevated levels of endothelial-derived EVs are observed as early as two weeks after HTN onset and may be clinically meaningful biomarkers. Further, T-cells have been implicated in the development of HTN, and T cell-derived EVs are also elevated in arterial HTN and correlate with blood pressure levels. EVs from kidney tubules also play functional roles in HTN and have been shown to carry functional units of sodium transporters; urinary EVs containing these kidney-derived EVs may serve as liquid biopsy for HTN as they show changes in protein expression in the kidney over time.

Next, Dirk Dittmer (University of North Carolina Chapel Hill) presented recent work on the use of super-resolution imaging of single EVs and their protein components. Using a four-step EV isolation pipeline, Dr. Dittmer’s group can generate approximately 10^13^ EVs per week that express the canonical EV markers and are cell fusion competent in culture. Further, these EVs can also be taken up *in vivo* in a non-human primate model to study biodistribution. Some important questions to consider when studying EV composition are whether EV membranes are homogenous or if they have lipid microdomains; if two tetraspanins can be present on the same EV or if there are just mixed populations of multiple types of EVs; and if two tetraspanins can be located on the same EV, are they present within the same microdomain? CryoEM reveals that EV morphology is mostly round with protein protrusions; super-resolution microscopy (dSTORM) strives to visualize these EVs and the protein composition of their microdomains. dSTORM data shows similar size profiles to nanoparticle tracking analysis, while also revealing that EVs have local membrane domains through fluorescent labeling of the EV membrane and the CD81 tetraspanin. While the technological advances in this area are encouraging, more work is needed to allow for the delineation between multiple proteins on the surface of a single EV.

Setty Magana (Nationwide Children’s and Ohio State University) shared recent work on the role of EVs and non-coding RNAs in multiple sclerosis (MS) across the lifespan. MS onset typically occurs around 20-40 years of age, but 3%-10% have childhood or adolescent onset, termed pediatric MS; this leads to earlier and higher relapse rates, early cognitive impairment, and brain atrophy. While there is shared pathophysiology between pediatric and adult MS, adult observations cannot be extrapolated to pediatric cases. Although the etiology is unknown, obesity and elevated body mass index (BMI) in genetically susceptible individuals are associated with an increased risk of developing pediatric MS. Adipose-associated cytokines (adipokines) and chronic low-grade metabolic inflammation are implicated in both pediatric and adult MS, and adiponectin positive EVs were detected in the serum from both MS patient populations. Interestingly, these EVs had distinct miRNA profiles between active and stable MS, as well as adult versus pediatric MS, with some identified miRNAs implicated in blood-brain barrier dysfunction. There is a need for the development of a precision neuroimmune EV-omics pipeline that would allow for biomarker discovery and individualized therapeutics.

The next speaker was Heather Branscome (American Type Culture Collection; ATCC), who spoke about large-scale EV manufacturing and its functional assessment. There is a strong need for EV reference materials, and ATCC has vesicles from both cancerous, non-cancerous, and stem cell lines that are available to the research community. Their EV manufacturing pipeline is highly reproducible with low lot-to-lot variability, robust quality standards, and high EV stability for up to 3 years. Interestingly, cancer and stem cell-derived EVs were profiled and were shown to have overlapping proteomes, but differential mapping of RNA to chromosomes. The functional properties of these EVs have also been studied with *in vivo* mouse experiments which show that EVs administered subcutaneously after a punch wound have healing abilities in a dose-dependent fashion. Further, the functional effects of EVs on 3D cellular models of repair are also being studied.

Haiying Zhang (Cornell University) gave the next presentation on the identification of distinct subsets of EVs and other nanoparticles and their role in cancer. Asymmetric flow field-flow fractionation (AF4) is a technique for separating EVs and from fluids; in this work, a heterogeneous sample was separated via AF4 and revealed three distinct populations of extracellular nanoparticles: exomeres (< 50 nm), small exosomes (50-80 nm), and large exosomes (90-120 nm). Proteomic analysis showed differences in protein expression in the three nanoparticle subsets, and *in vivo* biodistribution studies show uptake of all three in various organs. Distant primary tumors were shown to alter liver metabolism in multiple murine models, and interestingly, tumor-derived nanoparticles were shown to contribute to fatty liver generation and decreased drug metabolism in the liver. However, ablation of Rab27a diminishes tumor-induced fatty liver formation.

The last speaker of this session was Leif Anderson (Unchained Labs), who spoke about the ExoView EV characterization platform, which analyzes single vesicles to provide data on size, concentration, and tetraspanins profiles. Fluorescent measurements allow for multiplex analysis of samples and the identification of single proteins on single EVs. EV concentrations and subpopulations can be profiled based on the number of events bound to a specific antibody spot on the ExoView chips and up to five biomarkers can be simultaneously assayed at the same time. Captured vesicles can also be permeabilized to allow researchers to determine if specific proteins are on the inside or outside of the EV.

The second morning session was on EVs and the CNS and was moderated by Avi Nath (NIH) and Norman Haughey (Johns Hopkins University).

The keynote speaker for this session, Julie Saugstad (Oregon Health & Science University), gave a presentation focusing on the effects of APOE genotype and sex on cerebrospinal fluid (CSF) EVs in Alzheimer’s disease (AD). AD is the most common form of dementia and affects 6.2 million Americans over the age of 65. While the etiology of AD is unknown, mutations in the apolipoprotein E-e4 (APOE4) variant may play a key role in the development and pathophysiology of the disease. By the time AD is discovered, it is too late, and there are currently no effective therapies to treat AD; as such, there is a massive need for early diagnostic biomarkers and therapeutic interventions. Currently, increases in CSF amyloid beta-42 (Aβ42) levels with concurrent decreases in tau can sometimes be used as a diagnostic tool for identifying AD, but better biomarkers are needed. miRNA profiling from CSF revealed 36 miRNAs that differentiate AD from control patients, with slight increases in performance when APOE4 genotype and Aβ42:tau ratio are also considered. The longitudinal stability of miRNAs is also being considered, as patients typically present with mild cognitive impairment (MCI) prior to progressing into AD, and being able to predict which patients will progress to AD has high clinical relevance. Understanding the plasma profiles of potential miRNA biomarkers is also extremely important because plasma is much easier to obtain than CSF. CSF EV miRNAs were also profiled and found AD-associated miRNAs in EVs, and when sex is considered, differential expression of specific miRNAs is observed in males versus females. Future work aims to characterize EV surface proteins in AD and other neurodegenerative diseases and better understand how the expression of various AD-associated miRNAs is regulated.

The next speaker for this session was Tsuneya Ikezu (Mayo Clinic Florida), who shared recent work on microglial EVs. Following the tau propagation mechanism, these microglial EVs have been shown to spread tau pathology in AD. As extracellular aggregates of Aβ negatively impact synapse function, microglia begin to take up tau and release it in EVs and spread disease pathology in the AD brain. To identify genes necessary for microglial EV release, the Ikezu lab performed a genome-wide screen from cultured murine microglia and validated potential targets *in vitro* with primary microglia and siRNA. When microglia are stimulated with ATP or lipopolysaccharide (LPS), they are transformed into a proinflammatory state and release EVs. However, siRNA-based silencing of several genes inhibited ATP/LPS-induced EV release from microglia. These genes may be important targets for reducing microglial-EV tau propagation in diseases like AD.

Travis Thompson (UMass Chan Medical School) presented next on the potential role of EVs in the regulation of synaptic plasticity. A large majority of the genome appears to be viral-like and contains retrotransposons. In the drosophila, the neuromuscular junction is a tractable interface and the pre- to postsynaptic transfer of dArc1 retrotransposon-like transcript can be observed. The transfer of which behaves like a capsid and the 3’ UTR is necessary and sufficient for transfer. The transposon, Copia, is also present in neuromuscular junction EVs. If it is knocked down, the synapse is expanded and about 50%-60% more synaptic boutons are formed; however, they are abnormal. Interestingly, there appears to be a balance between dArc1 and Copia in that the knockdown of one results in the upregulation of the other. They also appear to be mutually exclusive in EVs; understanding the expression of these two genes and how they affect synaptic plasticity warrants further investigation.

The next presenter was Gagan Deep (Wake Forest School of Medicine), whose work focused on plasma BDEV-miRNAs. Many studies have focused on total or neuron-derived EVs, but other BDEVs may play important roles in disease pathology and may serve as blood-based biomarkers for AD. Total EVs were isolated from blood plasma and further separation based on protein markers associated with specific cell types (neurons, astrocytes, microglia, oligodendrocytes, endothelial cells, and pericytes) was performed. Several miRNAs associated with pericyte and endothelial-derived EVs were able to distinguish control, MCI, MCI conversion to AD, and AD with high accuracy, suggesting vascular impairment may be implicated in these diseases.

Norman Haughey (Johns Hopkins University) gave the last presentation of the morning; this talk focused on the clearance of Aβ into peripheral circulation, which activates an innate immune response and induces leukocyte transmigration into the brain. ADEVs released in response to IL-1β or ATP stimulation alter the functionality of recipient neurons by promoting the translation and amyloidogenic processing of amyloid precursor protein. Interestingly, these ADEVs can leave the CNS and communicate with the periphery, which may indicate a mechanism through which the brain signals inflammation. For example, in a mouse model of AD (APP/PS1), total brain EVs are reduced, while circulating plasma EVs are increased.

The first afternoon session was a continuation of talks focusing on EVs and the CNS and was moderated by Dirk Dittmer (University of North Carolina Chapel Hill) and Ramin Hakami (George Mason University).

The keynote speaker for this session was Navneet Dhillon (University of Kansas Medical Center), who gave a presentation on circulating EVs in long COVID-19. Post-acute sequelae of COVID-19 (PASC), commonly referred to as ‘long-COVID’, occurs in a subset of patients and has a wide range of symptoms, but key physiological changes that occur after COVID-19 infection including endothelial injury, vascular remodeling, and coagulopathy, likely participate in PASC pathogenesis. As EVs play major roles in inflammatory responses and regulation of vascular function, it is likely that they are implicated in PASC. EVs isolated from asymptomatic, moderate (no oxygen), moderate (oxygen), and severe COVID-19 patients revealed higher levels of proinflammatory, thrombosis, and coagulation markers in sEVs from severe COVID-19 patients. EVs from critically ill patients also induce pulmonary microvascular endothelial injury in an *in vitro* model. The SARS-CoV-2 spike protein itself can cause endothelial damage, while viral RNA can induce a proinflammatory response. The spike protein and viral RNA appear to persist in the circulation of PASC patients, and circulating sEVs from these patients were found to have the spike protein present on their surface.

The next presenter of this session was Sowmya Yelamanchil (University of Nebraska Medical Center), whose talk focused on the role of mitochondrial-derived vesicles (MDVs) in HAND. MDVs, also termed mitovesicles, are heterogenous and may play key roles in mitochondrial quality control and allow for crosstalk between organelles within a cell. MDVs were isolated and characterized from the brains of a transgenic rat model of HIV and found reduced expression of mitochondrial complex proteins in the transgenic MDVs relative to controls. It is possible that these MDVs reflect pathological changes in mitochondria or that they themselves may contribute to disease pathology.

Xian Shuang Liu (Henry Ford Health) gave the next talk on the role of sEVs and their miRNAs on post-stroke neurogenesis and angiogenesis. Stroke-induced angiogenesis is coupled with neurogenesis and contributes to spontaneous functional recovery as neuroblasts migrate along blood vessels to reach peri-infarct regions of the brain. *In vitro* experiments demonstrate that sEVs from ischemic cerebral endothelial cells (CECs) deliver miRNAs to neural stem cells (NSCs) and enhance their proliferation and differentiation. NSC-EVs increase capillary tube formation and migration of recipient CECs by transferring miRNAs. Interestingly, inhibition of EV release impairs miRNA transfer, both from CEC-EVs to recipient NSCs and from NSC-EVs to recipient CECs. Further, in an animal model CEC-EVs cross the blood-brain barrier and improve post-stroke recovery as assessed via several behavioral assays.

Next, Christie Fowler (University of California, Irvine) presented work on the characterization of EV release in the prefrontal cortex and nucleus accumbens. There are inherent differences between the way cells and the EVs they release interact *in vivo* versus *in vitro.* In the brain, cells and their released EVs interact with other populations of neighboring cells, whereas in culture, the extracellular environment is less enriching. To better understand EV dynamics *in vivo*, a transgenic CD81NeonGreen mouse line was crossed with a transgenic dopamine mouse line so that EVs from dopaminergic neurons could be monitored. Super-resolution microscopy revealed EVs in the ventral tegmental area, which is part of the reward pathway; when nicotinic receptors on the surface of the presynaptic terminal were activated with nicotine, an increase in EV release was observed.

The last speaker for this session was Navneet Dogra (Icahn School of Medicine at Mount Sinai), who shared recent work on characterizing the neurosecretome. Brain tissue was gently dissociated and EVs were isolated from the extracellular space via SEC. Each fraction was profiled based on its size and protein concentration to determine which fractions likely contain cellular debris and microvesicles, sEVs, and extracellular proteins, nucleic acids, and nucleic proteins. Diverse RNA profiles show distinct clustering of extracellular proteins and nucleic acids, sEVs, and cell debris.

The second afternoon session of Day 2 was on Cancer and EVs and was moderated by Lance Liotta (George Mason University) and Meta Kuehn (Duke University).

The keynote speaker for this session was Aurelio Lorico (Touro University Nevada), who presented work on targeting spathasome-based nuclear transport for cancer and viral therapy. Rab7+ late endosomes come together with nuclear envelope invaginations (NEIs) to form spathasomes, which are newly discovered cellular organelles that deliver biological information from EVs to the nucleus. Both HeLa and T cells typically have round nuclei; however, after exposure to HIV or EVs, NEI is activated, likely to deliver cargo to the cell’s nucleus. Endosomes arrive at the NEI and interestingly have different NEIs depending on what they were exposed to, and the type of NEI that is formed correlates with productive infection. The molecular interactions between late endosomes and NEIs rely upon the VOR complex, VAP-A, ORP3, and Rab7, allowing tethering of the late endosome to the NEI. Interestingly, the knockdown of VAP-A or ORP3 inhibits the transport of CD9 to the nucleus.

Diloram Sass (National Cancer Institute/NIH) presented next on the use of EVs as biomarkers of clinical outcomes in cancer and brain injury survivors. EVs are being utilized as prognostic tools for cancer and neurodegenerative diseases, but very few groups utilize EVs as potential biomarkers of clinical outcomes, especially patient-reported outcomes. Hierarchical clustering revealed differential expression of some protein markers in the soluble versus EV fraction from cancer patients. Further, EV-associated cytokines correlated with symptom burden reported by these patients. EVs isolated from traumatic brain injury patients with post-traumatic stress disorder revealed higher levels of neurofilament light chain, further suggesting that patient-reported outcomes correlate with differences observed in EV profiles.

Next, Jennifer Finan (Oregon Health and Science University) spoke about pancreatic ductal adenocarcinoma (PDAC) EVs. Two major struggles with PDAC are early detection and response to therapy; typically, by the time the cancer has been detected, it is too late for effective therapy. This cancer is supported by noncancerous cells in the tumor microenvironment, and these cells have been shown to uptake PDAC EVs, which may induce a pro-tumorigenic phenotype. Cancer-associated fibroblasts were especially shown to uptake PDAC EVs *in vitro*, relative to uptake by other PDAC cells and normal epithelial and endothelial cells. EV uptake can also be monitored *in vivo* by using the PalmGRET reporter in a mouse model of PDAC to determine which cells in the tumor microenvironment take up PDAC EVs.

Olivier Loudig (Center for Discovery and Innovation) was the next speaker in this session and presented novel findings on exhaled EVs (exh-EVs). EVs present in breath condensate exhaled from the lungs may provide important information about the health and physiology of the lung. Within just 10 minutes of breathing, 2 mL of liquid can be obtained with a specialized device. Various techniques for isolating these EVs were utilized, and it was found that magnetic beads hinder the discovery of circulating sEV-based miRNA biomarkers and non-specifically capture EVs. Instead, a novel method was developed termed EV-CATCHER(Extracellular Vesicle Capture by AnTibody of CHoice and Enzymatic Release) to capture EVs from the exhaled breath condensate. These exh-EVs have significant potential to serve as biomarkers for the detection of lung cancer, as differences in miRNA profiles were detected relative to healthy age-matched controls.

The next presenter was Bojan Losic (Guardant Health), who gave a talk focusing on the use of unannotated small RNA clusters in circulating EVs as biomarkers for early-stage liver cancer. Hepatocellular carcinoma (HCC) is the most dominant form of liver cancer and can be caused by chronic hepatitis and cirrhosis and patients with these conditions are good candidates for surveillance. Small RNA in plasma EVs sequencing reveals that most hg38 alignments tile to unannotated loci; therefore, the strategy was to utilize data-driven small RNA gene quantitation to classify and identify EV RNA de novo alignment patterns. Small unannotated RNAs are strongly expressed in high-risk patients, and this unannotated EV RNA signature performed better than the current clinical standard in early HCC detection. Additionally, a new point-of-care liquid biopsy platform was developed to quickly quantify EV RNAs from low-volume clinical samples to help identify HCC patients.

Xiaoli Yu (University of Colorado Anschutz Medical Campus) presented next on glioblastoma EV-specific peptides. Glioblastoma is the most common and lethal primary brain tumor and its diagnosis relies on imaging and postoperative pathological diagnosis. Proteomic analysis revealed that glioblastoma EVs possess unique features, and the use of an unbiased approach for EV identification may provide a way to see the surface of EVs that do not necessarily express the typical tetraspanins. Glioblastoma EVs derived from patient plasma as well as from a cell line were shown to kill neuronal cells. Interestingly, when specific peptides were blocked, EV-induced neuronal death was inhibited.

The last presenter for this session was Guoku Hu (University of Nebraska Medical Center), who shared recent work on ADEVs and their effects on neuronal primary cilia in morphine tolerance. Opioid overdose is the leading cause of accidental death and opioid tolerance increases the risk of overdose death. The primary cilium is a singular organelle within cells that can detect extracellular cues and play a role during development and tissue homeostasis. Interestingly, changes to the primary cilia number and length have been implicated in drug resistance in cancer, and morphine exposure promotes primary ciliogenesis in neurons. Morphine exposure also increases the release of EVs from astrocytes, and these morphine-induced ADEVs promote primary ciliogenesis in neurons. Interestingly, inhibition of EV release or primary ciliogenesis prevents morphine tolerance in mice.

At the conclusion of this session, an NIH grant writing workshop facilitated by Fatah Kashanchi (George Mason University), Avi Nath (NIH), and Philip Askenase (Yale School of Medicine) was held. This workshop focused on how the grant review process typically proceeds and had an interactive component in which participants worked to write a specific aims page as a group during the session. The grant writing workshop was followed by a poster session.

**Day 3 of Meeting:** The morning of the third day of the conference began with a session on Infectious Diseases and was moderated by Christie Fowler (University of California, Irvine) and Sowmya Yelamanchil (University of Nebraska Medical Center).

The keynote speaker for this session was Meta Kuehn (Duke University), who presented work on cargo selectivity of bacterial EVs. Both gram-positive and gram-negative bacteria produce EVs with a wide variety of functions, determined by their wide variety of cargo, which can be impacted by changes in environmental conditions. Additionally, the SigmaE stress pathway can also alter EV release and cargo composition by preferentially packing specific proteins into EVs. Further, the physical tethering of various proteins can also play a role in whether a protein is incorporated or excluded from an EV. Mechanisms of cargo enrichment and sorting can further our understanding of both general biology and application-based research.

Ramin Hakami (George Mason University) gave the next presentation on the regulation of EV release during infection with cytoplasmic RNA viruses. Rift Valley fever virus (RVFV) is an endemic RNA virus that is predominately located in Sub-Saharan Africa and is typically transmitted via mosquito bites. This disease results in hemorrhagic fever, neurological impairments, blindness, and liver failure. Cells infected with this virus produce both EVs and viral particles, which can be separated with density gradient centrifugation. Cells pretreated with EVs isolated from cells infected with RVFV and then infected with the virus had reduced viral loads, indicating a protective effect from these EVs. It is hypothesized that this anti-viral effect is mediated through the IFN pathway and activation of autophagy.

The next presentation was given by Jay Debnath (University of California San Francisco), whose presentation focused on the role of autophagy in EV cargo selection and release. Autophagy involves the activation of LC3/ATG8 proteins, and LC3 plays an important role in capturing cargo to package into autophagosomes. Activation of the secretory autophagy pathway leads to the incorporation of proteins lacking an N-terminal ER signal sequence into autophagosomes, which are then released from the cell, as opposed to fusing with the lysosome. The LC3-dependent EV loading and secretion (LDELS) pathway enables secretory autophagy through EVs, primarily carrying RNA binding proteins and endosomal transmembrane proteins such as the transferrin receptor. Interestingly, inhibition of lysosomal acidification promotes the release of EVs and/or particles through the secretory autophagy during lysosome inhibition (SALI) pathway. LEDLS and SALI are two distinct secretory autophagy pathways that can result in EV release from cells.

Sergey Iordanskiy (Uniformed Services University of the Health Sciences) presented next on recent work focusing on the impacts of endogenous retroviruses on macrophage differentiation and their effect on bystander cells. Activation of monocytes and macrophages under conditions of stress leads to an increase in the expression of IFN-1, interferon-stimulated genes, chemokines, and proinflammatory cytokines, and some of these cytokines are localized in EVs. Exposure to radiation-induced stress, pathogen-associated molecular patterns, and cytokines upregulates transcription of several human endogenous retroviruses in monocytes and macrophages. These human endogenous retroviruses may then amplify the inflammatory response in these cells and mediate changes in the EVs they release.

Next, Yisel Cantres-Rosario (University of Puerto Rico Medical Sciences Campus) gave a presentation on the association of soluble insulin receptor levels in plasma EVs and HAND. Diabetes and insulin resistance are common comorbidities for PLWH, and these metabolic disorders are associated with an increased risk of neurocognitive impairment and dementia. PLWH who have symptomatic cognitive impairment have elevated levels of soluble insulin receptors in plasma and CSF relative to PLWH who are not cognitively impaired, as well as HIV- controls. Interestingly, BDEV levels are elevated in the plasma of PLWH and correlate with EV-associated reactive oxygen species.

The last talk of this session was given by Nazar Filovio (Alpha Nano Tech), which focused on the isolation and characterization of EVs. Commercially available standards may be a useful tool for researchers to implement in their experiments to aid in quality control.

The second morning session entitled “Technology and Therapeutics” was moderated by Ashley Russell (Penn State Erie) and Sergey Iordanskiy (Uniformed Services University of the Health Sciences).

The keynote speaker for this session was David Walt (Harvard Medical School), whose talk focused on ultrasensitive digital technologies for measuring EVs. The overarching goal of this work is to identify EVs from any tissue or organ by capturing them on a magnetic bead with a capture antibody, then utilizing detector antibodies to probe for the presence of specific proteins on the captured EV. Additionally, if vesicles are treated with proteinase K to remove all extracellular/surface proteins, the vesicles can then be burst, and their internal cargo assessed. The single molecule array assay allows individual EVs to be captured and assessed at the single-molecule level. Through this, it was found that Ras is a universal protein in circulating EVs and is highly enriched in the plasma of pancreatic cancer patients compared to healthy controls. This new technology holds promising diagnostic potential for a number of diseases through the assessment of single EVs.

The next talk was given by Reka Haraszti (University Hospital Tubingen, Germany), whose presentation focused on the use of sEVs for the delivery of siRNA to neurons and immune cells. Cholesterol’s hydrophobic properties play a crucial role in anchoring siRNA to EV membranes. Conjugating siRNA to lipids enables efficient incorporation into EVs, making them promising carriers. EVs over or underloaded with siRNA do not have strong effects on target mRNAs in recipient cells; approximately 3,000 siRNAs per EV seems to be the ideal amount for loading and delivery. Cells that are stressed via serum starvation release EVs with altered protein and lipid compositions and are better at delivering siRNAs than EVs derived from non-stressed cells.

Elena Batrakova (University of North Carolina at Chapel Hill) gave the next talk on the development of biomimetic drug delivery systems for the treatment of neurodegenerative disorders. EVs are thought to be natural drug carriers that enable long-distance intercellular communication by facilitating the transfer of proteins and functional nucleic acids with high stability and biocompatibility and low toxicity. As such, EVs can deliver drug-loaded nanoparticles to recipient cells very efficiently. The route of administration is an important consideration when determining how to administer EVs, as intrathecal, intraperitoneal, intravenous, and intranasal all result in slightly different uptake patterns throughout the body. For example, intranasal administration of EVs in mice resulted in greater uptake in the brain relative to intravenous administration, while intrathecal administration of EVs in non-human primates resulted in greater CNS uptake compared to intraperitoneal and intravenous administrations. Interestingly, intrathecal administration of peripheral blood mononuclear cells into the non-human primate model resulted in more CNS uptake than the EVs.

Next, Chulee Choi (ILIAS Biologics Incorporated) shared recent advances in the development of new strategies for loading cargo into EVs and compared various techniques, including passive loading, anchored cargo loading, and non-anchored cargo loading. Using a Cre-based reporter system, it was found that non-anchored Cre loaded into EVs resulted in the highest amount of biodistribution relative to anchored Cre. Surface engineering of EVs was also found to enhance the targeted delivery of EVs to specific tissues.

Yuntao Wu (George Mason University) presented next on the involvement of the actin cytoskeleton in viral and EV uptake. The actin cytoskeleton plays a fundamental role in cells by providing structural support, facilitating cell movement, and participating in various cellular processes. Viral infection can trigger remodeling of the cortical actin underlying the cell’s surface and promote viral entry into the cell. For example, the knockdown of ARP2/3 impairs HIV-1 viral uptake, while actin-stimulating peptides can promote HIV-1 infection. These principles can also be applied to the cellular uptake of EVs and warrant further study.

The last presentation of the conference was given by Jia Wang (Oxford Nanoimaging), who shared information on the ONI Nanoimager. This equipment allows for super-resolution imaging of both live cells and EVs on the benchtop. Its four lasers allow for multiplex analysis and imaging of EV biogenesis, release and transport, uptake, and assessment of EV cargo.

The session concluded with a prize distribution ceremony for the best oral & poster presentations. The recipients of the best oral presentations were Navneet Dogra (Icahn School of Medicine at Mount Sinai), Carlos Diaz-Osterman (Ponce Health Sciences University), and Ursula Sandau (Oregon Health & Science University). The best poster presentation awards went to Sameh Almousa (Wake Forest School of Medicine), Tzu-Yi Chen (Icahn School of Medicine at Mount Sinai), Edgar Gonzalez-Kozlova (Icahn School of Medicine at Mount Sinai), and Sarah Baker (Oregon Health & Science University). Aurelio Lorico (Touro University Nevada) received an award for the most innovative talk award.

The meeting provided an informative and comprehensive overview of the EV field, with work spanning from cell culture and animal model-based studies to cutting-edge clinical research. Notably, advancements in understanding the mechanisms by which EV biogenesis, cargo loading, and uptake are controlled were a common theme throughout the meeting. Additionally, state-of-the-art techniques for visualization and characterization of EVs at the single EV level, as well as their biodistribution *in vivo* were also heavily discussed. The clinical translational potential of EVs was highlighted at this meeting, with several presentations focusing on the use of EVs as biomarkers for neurodegenerative disorders and various cancers, as well as their use as engineered drug delivery vehicles. Overall, this meeting provided a space for EV enthusiasts to share novel, exciting work, exchange ideas, and forge new collaborations with the hopes of paving the way for future advancements in our understanding of basic and translational EV biology.

ASIC would like to thank the Organizing Committee for organizing this meeting, especially given the continued use of the hybrid format, which allowed for enhanced scientific exchange. Special thanks are also extended to Gwen Cox for her commitment to ensuring this meeting ran smoothly.

Fatah Kashanchi, George Mason University

Leonid Margolis, National Institute of Child Health and Human Development, National Institutes of Health

Julie Saugstad, Oregon Health & Science University

Meta Kuehn, Duke University

Michael Graner, University of Colorado Anschutz

Janusz Rak, McGill University

## DECLARATIONS

### Copyright

© The Author(s) 2023.

